# Supplementary material for: Development and characterization of an oral microbiome transplant among Australians for the treatment of dental caries and periodontal disease: A study protocol
Source: PLoS One. 2021 Nov 29;16(11):e0260433. doi: 10.1371/journal.pone.0260433 (PMC8629173; doi:10.1371/journal.pone.0260433)
Supplement: S1 File — This would be distributed to every eligible study participant informing about the study. Consent would be obtained after the information sheet has been completely read and understood by the participant. (DOCX) [file pone.0260433.s001.docx]

**PARTICIPANT INFORMATION SHEET**

**PROJECT TITLE:** Developing Oral Microbiome Transplants in Australia

**HUMAN RESEARCH ETHICS COMMITTEE APPROVAL NUMBER: H-2020-34609**

**PRINCIPAL INVESTIGATOR:** Dr. Peter Zilm and Dr. Laura Weyrich**.**

**STUDENT RESEARCHERS:** Dr. Sonia Nath and Kevin Ketagoda

**STUDENT’S DEGREE:** Doctor of Philosophy (PhD)

Dear Participant,

You are invited to participate in the research project described below.

**What is the project about?**

In the mouth, microbes (e.g. bacteria and fungi) play key roles in oral diseases, such as dental caries (tooth decay), periodontal (gum) disease, gingivitis, halitosis (bad breath) and oral cancers. The causes of caries and periodontal disease are directly linked to the microbes, such as bacteria, present in dental plaque. The microbes contained within plaque are notoriously difficult to remove using classical methods, such as tooth brushing or antibiotics.

In the lower gastro-intestinal tract, a procedure known as Fecal Microbiota Transplant transfers microbiota from healthy individuals into the bowels of people suffering from acute intestinal infections to override the existing microbes and improve gut health. Microbial transplants do not currently exist for the mouth. For the first time, Oral Microbiota Transplantation (OMT) therapy will be examined as a new method to improve oral health by growing microbes from healthy donors in the lab and seeing if OMT will help improve and treat cavities and periodontal disease in mouse and rat models.

We are collecting microbes from the mouths of healthy donors. We will describe the diversity of these microbial communities and groups and explore how these microbes grow in the laboratory (Aim 1). We will then develop a suitable vehicle and method to transfer oral microbes (Aim 2). Finally, we will examine the safety and efficacy of OMT in rodent laboratory models to treat caries and periodontal disease (Aim 3).

**Study Aims:**

**Aim 1: Characterize oral biofilm growth using a novel *in vitro* model**

a) Describe the oral plaque microbes of healthy donors.

b) In the laboratory, improve growth conditions for OMT donor material.

c) Examine responses of healthy oral microbes to alternative lifestyle factors.

d) In the laboratory, test the competition between different bacteria in the mouth and determine if OMT can work*.*

**Aim 2: Develop a delivery system for OMT therapy**

a) Test microbial survival and efficient transfer or delivery of plaque microbes using different mediums (*e.g.* hydrogels, dental varnish or mouthwashes).

**Aim 3: Test oral microbiota transplants in rodent models**

a) Examine safety and efficacy of OMT of human microbiota into healthy rodents.

b) Examine safety and efficacy of OMT in an animal disease models.

This study will provide the foundational data necessary to determine if and how human OMT clinical trials should proceed in the future. As this proposed research is entirely original, there is no guarantee that a clinical trial will arise following this research.

**Who is undertaking the project?**

This project is being conducted by Dr. Peter Zilm, Lisa Jamieson and Kostas Kapellas at the University of Adelaide and Dr. Laura Weyrich at Pennsylvania State University, U.S.A. Aspects of this research will also form the basis for the degree of Doctor of Philosophy for two University of Adelaide students, Sonia Nath under the supervision of Dr. Peter Zilm, Lisa Jamieson and Kostas Kapellas, and Kevin Ketagoda under the supervision of Dr. Peter Zilm and Laura Weyrich.

The Australian National Health and Medical Research Council have funded this project.

**Why am I being invited to participate?**

You are being invited to participate as you have identified yourself as being physically healthy without chronic diseases and critically, have no oral conditions such as tooth decay or gum disease. The latter will need to be confirmed via an oral assessment by research personnel in order for you to be included in this study.

**What am I being invited to do?**

Your participation is entirely voluntary and will involve a single session where you will be invited to:

- Complete a questionnaire that will collect demographic (e.g. age, sex), health (general (e.g. exercise and physical activity), oral health behaviours (e.g. tooth brushing, use of chewing gum), lifestyle (e.g. smoking and alcohol consumption) and dietary information (e.g. what foods you commonly eat, whether you take any probiotics (i.e. Yakult or Kombucha), whether you are on a diet), and medical history to determine if you have any pre-existing medical conditions or are taking any medications.
- For your convenience, the questionnaire, oral assessment, saliva and plaque sampling will be conducted at your home or other convenient location.
- Undergo an oral assessment that will collect information about the number of teeth, tooth decay, gum disease, oral infections and other conditions (e.g. pathology)
- Sampling of dental plaque from the lip-side of an upper and lower front tooth as well as from the check surfaces of the upper and lower molars.
- Provide a 2 mL sample of saliva in a test tube.

**What will happen with my samples?**

Collected plaque and saliva samples will be transported to The University of Adelaide’s, Oral Microbiology Laboratory located in the Helen Mayo South Building, Frome Road, Adelaide and stored in a -20^o^C freezer. The laboratory work assessing dental plaque samples will involve culturing (growing) the samples in the first stage. Following this, we will describe the DNA from microbes in the sample by extracting the DNA and sequencing it via a process known as High Throughput DNA Sequencing (HTS). No human DNA will be analyzed in this study.

All the samples along with their aliquots will be frozen and kept securely at The University of Adelaide’s Oral Microbiology Laboratory which is only accessible via secured swipe-card. Saliva and plaque samples collected from participants will be disposed of at the conclusion of the study. Microbes grown from the samples will be maintained in perpetuity to develop OMT in the future.

Analysis of microbiome data will be conducted in the laboratory of Dr. Weyrich at Pennsylvania State University, U.S.A. as their research group are global experts in this area. This analysis will involves creating microbial DNA libraries and High Throughput DNA Sequencing (HTS) of samples to examine the microbes present from each sample. No human DNA will be analysed during this process.

**How much time will my involvement in the project take?**

It is envisaged that your total involvement will take approximately 60 minutes to complete all parts of the study. We expect that completing the health and lifestyle questionnaire will require no more than 15 minutes whilst the oral assessment and plaque sampling will require up to 25 minutes.

**Are there any risks associated with participating in this project?**

There are no foreseeable risks in your participation in this study. Some people with gum disease may experience mild discomfort when undergoing an oral assessment. However, as this study aims to recruit people with a healthy mouth, it is not expected that any study participants will experience this.

**What are the potential benefits of the research project?**

Beyond possibly gaining a better understanding of your oral health status, there may be no immediate benefit in your participation in this study. This is research may provide crucial information that will aid in our understanding of which oral microbes can be transplanted in the mouth to modify a microbiome from a diseased state to one conducive to health. As this study in novel, it cannot be guaranteed that such information will be attained.

**Can I withdraw from the project?**

Participation in this project is completely voluntary. If you agree to participate, you can withdraw from the study at any time without having to provide a reason for doing so. If you choose to withdraw, you may elect to request that all or only parts of your data collected can be kept for study purposes, or removed entirely.

**What will happen to my information?**

*Future research:* The present study will examine the feasibility of OMT in an animal model. If it is shown to be feasible, it is planned that future studies may be conducted involving humans. Samples collected in the present project may be used as part of this future research projects. Therefore, extended consent will be obtained permitting for the use of data and microbiome samples in future research projects that will arise as an extension of the original project.

*Confidentiality and privacy:* Data forms including your name and contact details will be collected as part of this study along with a participant ID. The participant ID will be recorded on all data forms namely the questionnaire and oral assessment forms in addition to tubes containing your dental plaque samples. Only the research team members involved in your data collection appointment will know your name and study participant ID. Following collection, any paperwork containing your name (i.e. consent form) will be separated from the remaining data forms containing only your participant ID and stored in a separate locked file storage cabinet for five years. The utmost care will be taken to ensure that no personally identifying details are revealed. All analysis of data and plaque samples arising from your study appointment will be de-identified.

*Publishing:* Reporting of this study will be via several avenues including scientific journal articles and plain language reports which will be provided to study participants, conference presentations, PhD theses and presentation within the universities of investigators. All analysis and interpretation of study findings will be in aggregate meaning that no identifiable information will be presented. Your information will only be used as described in this participant information sheet and it will only be disclosed according to the consent provided, except as required by law.

**Who do I contact if I have questions about the project?**

All queries concerning the project should be directed to the study project manager Dr. Kostas Kapellas via telephone 08 8313 7339 or email [kostas.kapellas@adelaide.edu.au](mailto:kostas.kapellas@adelaide.edu.au).

Other research team members along with their contact details are listed below:

Dr. Peter Zilm, 08 8313 5676, peter.zilm@adelaide.edu.au

Assoc. Prof. Laura Weyrich, (814) 867-0016, lsw132@psu.edu

Prof. Lisa Jamieson 08 8313 4611, lisa.jamieson@adelaide.edu.au

Dr. Sonia Nath sonia.nath@adelaide.edu.au

Dr. Kevin Ketagoda, kevin.ketagoda@adelaide.edu.au

**What if I have a complaint or any concerns?**

The study has been approved by the Human Research Ethics Committee at the University of Adelaide (approval number H-2020-34609). This research project will be conducted according to the NHMRC National Statement on Ethical Conduct in Human Research 2007 (Updated 2018). If you have questions or problems associated with the practical aspects of your participation in the project, or wish to raise a concern or complaint about the project, then you should consult the Principal Investigator. If you wish to speak with an independent person regarding concerns or a complaint, the University’s policy on research involving human participants, or your rights as a participant, please contact the Human Research Ethics Committee’s Secretariat on:

Phone: +61 8 8313 6028

Email: [hrec@adelaide.edu.au](mailto:hrec@adelaide.edu.au)

Post: Level 4, Rundle Mall Plaza, 50 Rundle Mall, ADELAIDE SA 5000

Any complaint or concern will be treated in confidence and fully investigated. You will be informed of the outcome.

**If I want to participate, what do I do?**

To participate in the study, contact the study project manager Dr. Kostas Kapellas via telephone 08 8313 7339 or email [kostas.kapellas@adelaide.edu.au](mailto:kostas.kapellas@adelaide.edu.au) to book an appointment.

Yours sincerely,

Dr. Peter Zilm

Assoc. Prof. Laura Weyrich

Dr. Kostas Kapellas

Prof. Lisa Jamieson

Dr. Sonia Nath

Mr. Kevin Ketagoda
