# Supplementary material for: Development and characterization of an oral microbiome transplant among Australians for the treatment of dental caries and periodontal disease: A study protocol
Source: PLoS One. 2021 Nov 29;16(11):e0260433. doi: 10.1371/journal.pone.0260433 (PMC8629173; doi:10.1371/journal.pone.0260433)
Supplement: S2 File — The consent form would require patient’s signature on agreeing to participate in the study. (DOCX) [file pone.0260433.s002.docx]

**Human Research Ethics Committee (HREC)**

**CONSENT FORM**

1. I have read the attached Information Sheet and agree to take part in the following research project:

| **Title:** | **Developing Oral Microbiome Transplants in Australia** |
| --- | --- |
| **Ethics Approval Number:** | ***H-2020-34609*** |

1. I have had the project, so far as it affects me, and the potential risks and burdens fully explained to my satisfaction by the research worker. I have had the opportunity to ask any questions I may have about the project and my participation. My consent is given freely.
2. I have been given the opportunity to have a member of my family or a friend present while the project was explained to me.
3. Although I understand the purpose of the research project is to develop healthy oral microbiome samples with the intention of improving the quality of dental care and potentially healthcare more generally, it has also been explained that my involvement may not be of any direct benefit to me.
4. I agree to participate in the activities as outlined in the participant information sheet [please tick].
   1. Complete a questionnaire that will collect demographic, health, lifestyle and dietary information [ ]
   2. Undergo an oral assessment that will collect information about the number of teeth, dental decay, periodontal (gum) disease, oral infections and other conditions [ ]
   3. Sampling of dental plaque [ ]
5. I understand that I am free to withdraw from the project at any time and that this will not affect any aspects relevant to the management of my health, now or in the future.
6. I have been informed that the information gained in the project may be published in journal articles, a PhD thesis, and may be presented at national and/or international conferences.
7. I have been informed that in the published materials I will not be identified and my personal results will not be divulged.
8. I agree to my information being used for future research purposes as follows:

Research undertaken by these same researcher(s) Yes  No

Research undertaken by any researcher(s) Yes  No

1. I hereby provide ‘extended’ consent for the use of my data or dental plaque samples in future research projects that are:

- (i) an extension of, or closely related to, the original project: Yes  No
- (ii) in the same general area of research (for example, genealogical, ethnographical, epidemiological, or chronic illness research): Yes  No

1. I hereby provide ‘unspecified’ consent for the use of my data or dental plaque samples in any future research:

Yes  No

1. I understand my information will only be disclosed according to the consent provided, except where disclosure is required by law.
2. I am aware that I should keep a copy of this Consent Form, when completed, and the attached Information Sheet.

**Participant to complete:**

Name: Signature: Date:

**Researcher/Witness to complete:**

I have described the nature of the research to

*(print name of participant)*

and in my opinion she/he understood the explanation.

Signature: Position: Date:
