# Supplementary material for: Development and characterization of an oral microbiome transplant among Australians for the treatment of dental caries and periodontal disease: A study protocol
Source: PLoS One. 2021 Nov 29;16(11):e0260433. doi: 10.1371/journal.pone.0260433 (PMC8629173; doi:10.1371/journal.pone.0260433)
Supplement: S3 File — This is online questionnaire designed in REDCap. (PDF) [file pone.0260433.s003.pdf]

Oral Microbiome Transplant Screening

Oral microbiome transplantation (OMT) is a novel concept of introducing health-associated oral microbiota into the oral cavity of diseased patients. The premise is to alter a dysbiotic microbiota and to restore the ecological balance back to health. This study will assess the effectiveness, feasibility, and safety of oral microbiome transplant in vitro and in vivo (rodent) models for the prevention and treatment of dental caries and periodontal disease.

Please complete the survey below.

Thank you!

First name	________________________
Last name	________________________
Date of Birth	________________________
Email address	________________________
Contact number	________________________
Are you 18 years or above?	◯ Yes<br>◯ No
Are you pregnant or lactating?	◯ Yes<br>◯ No
Do you have any of the following systemic conditions (currently diagnosed); diabetes, cardiovascular disease (angina or heart disorder), Cancer, Respiratory disorder, bone or joint disorder, epilepsy, gastro-intestinal disorder (ulcerative collitis, Crohn's disease) or kidney dysfunction?	◯ Yes<br>◯ No
Are you taking any of the following drugs orally for the last 3 months; 1. Antibiotics, Antifungal, Antiviral or Anti-parasitic 2. Corticosteroids (Prednisolone, Flonase, dexamethasone, Flovent) 3. Cytokines or drugs that can stimulate your immune system 4. Methotrexate or other agents that suppress your immune system 5. Commercial probiotics (eg. Probiotic tablets or Yakult)?	◯ Yes<br>◯ No
Do you have bleeding gums either during brushing your teeth, eating or chewing hard food?	◯ Yes<br>◯ No
Are you having bad breath?	◯ Yes<br>◯ No
Did you experience pain in your tooth in the last 3 months?	◯ Yes<br>◯ No

Do you have more than 8 teeth missing from your oral cavity?	◯ Yes<br>◯ No
Did you have dental filing done or dental cleaning (scaling or polishing) done in the last 3 months?	◯ Yes<br>◯ No
Do you currently have cold, flu-like symptoms, fever, cough, shortness of breath or recent travel history interstate or internationally?	◯ Yes<br>◯ No
Have you ever had a positive test for SARS-CoV-2 (COVID-19)?	◯ Yes<br>◯ No

Basic Details

Please complete the survey below.

Thank you!

1) Name of the Interviewer.
(Enter "self" if you are filling it yourself") __________________

2) Date of Interview __________________

3) Gender
◯ Male
◯ Female

4) Postal Address with postcode __________________

Demographic details

Please complete the survey below.

Thank you!

Are you of Aboriginal or Torres Strait Islander origin?	◯ No<br>◯ Aboriginal<br>◯ Torres Strait Islander<br>◯ Aboriginal and Torres Strait Islander<br>◯ Don't know/Rather not say
In which country were you born	◯ Australia<br>◯ England<br>◯ New Zealand<br>◯ India<br>◯ Italy<br>◯ Vietnam<br>◯ Philippines<br>◯ Other
Please specify	__________________________________
Do you speak a language other than English at home?	◯ Yes<br>◯ No
What languages do you mainly speak at home	☐ English<br>☐ Aboriginal<br>☐ Mandarin<br>☐ Italian<br>☐ Arabic<br>☐ Cantonese<br>☐ Greek<br>☐ Vietnamese<br>☐ Hindi<br>☐ Other
Please specify	__________________________________
What is the highest level of schooling you have completed?	◯ Primary school (year 7 or less)<br>◯ Year 8<br>◯ Year 9<br>◯ Year 10<br>◯ Year 11<br>◯ Year 12<br>◯ Don't know/Rather not say

What is the highest qualification or level of education you have completed?	◯ Postgraduate degree level-University Masters degree or PhD<br>◯ Graduate diploma/Graduate certificate level-Graduate specialization after Bachelor degree<br>◯ Bachelor or Honors degree level<br>◯ Advanced diploma/Diploma level/Associate degree-CAE; Teachers College or Nursing; University diploma<br>◯ Certificate level 1, 2, 3, 4- Trade certificate/apprenticeship/vocational (eg TAFE, hairdressing)<br>◯ Other qualification (Non-award courses, etc)<br>◯ None completed but currently studying at University<br>◯ None completed but currently studying at TAFE/apprenticeship<br>◯ None completed but currently studying Secondary school<br>◯ Don't know/Rather not say
Are you studying full time or part time?	◯ Full time student<br>◯ Part time student<br>◯ Not studying at School/TAFE/Uni
How would you describe your current employment status?	◯ Full time (35 or more hours per week)<br>◯ Part time (less than 35 hour per week)<br>◯ Not employed<br>◯ Don't know/Rather not say
Are you currently...	☐ Retired<br>☐ Home duties<br>☐ Unemployed and looking for work<br>☐ A student<br>☐ Not employed and not looking for work<br>☐ Don't know/ Rather not say

Smoking

Please complete the survey below.

Thank you!

Have you smoked more than 100 cigarettes in your lifetime?	◯ Yes<br>◯ No<br>◯ Rather not say
Do you currently smoke cigarettes?	◯ Yes<br>◯ No
For how long have you smoked cigarettes? Enter how many years.	__________________________
On a usual day, how many cigarettes do you smoke?	__________________________
Are you a former cigarette smoker?	◯ Yes<br>◯ No
How long ago did you stop smoking cigarettes?	__________________________<br>(Enter how many years.)
For how long have you smoked cigarettes?	
For how long have you smoked cigarettes?	__________________________
For how long have you smoked cigarettes?	__________________________
On a usual day, how many cigarettes do you smoke?	__________________________
Do you chew tobacco chew tobacco?	◯ Yes<br>◯ No<br>◯ Rather not say
Which of the following best describes your non-tobacco substance smoking status?	◯ I currently smoke non-tobacco substances<br>◯ I don't smoke non-tobacco substances now but I used to<br>◯ I have never smoked non-tobacco substances

Alcohol

Please complete the survey below.

Thank you!

Do you drink alcohol?	◯ Yes<br>◯ No<br>◯ Rather not say
How often do you drink alcohol?	◯ Daily<br>◯ Weekly<br>◯ Monthly<br>◯ Never
How much alcohol do you drink per week?	◯ 20+ Alcoholic drinks<br>◯ 8-19 Alcoholic drinks<br>◯ 1-7 Alcoholic drink<br>◯ No alcoholic drink

General Health Status

Please complete the survey below.

Thank you!

Are you currently taking medication for anything?	◯ Yes<br>◯ No<br>◯ Rather not say
If yes, mention the name(s) and dose(s) of your current medication?	__________________________________
Which of the following best describes your non-prescription drug use?	◯ I currently use non-prescription drugs<br>◯ I don't use non-prescription drugs now but I used to in the past<br>◯ I have never used non-prescription drugs

Self-rated health

Please complete the survey below.

Thank you!

		Excellent	Very good	Good	Fair	Poor
1)	Would you rate your general health as	◯	◯	◯	◯	◯
2)	Would you rate your oral health as	◯	◯	◯	◯	◯

Physical Activity

Please complete the survey below.

Thank you!

Does your work involve vigorous intensity that causes large increases in breathing or heart rate like carrying heavy loads, digging OR construction work for at least 10 minutes continuously?

◯ Yes
◯ No

In a typical week, on how many days do you do vigorous-intensity activities as part of your work? Mention number of days.

____

How much time do you spend doing vigorous-intensity activities at work on a typical day?

____

Does your work involve moderate-intensity activity that causes small increases in breathing or heart rate like brisk walking OR carrying light loads for at least 10 minutes continuously?

◯ Yes
◯ No

In a typical week, on how many days do you do moderate-intensity activities as part of your work? Mention number of days

____

How much time do you spend doing moderate-intensity activities on a typical day?

____

Do you walk or use a bicycle (pedal cycle) for at least 10 minutes continuously to get to and from places?

◯ Yes
◯ No

In a typical week, on how many days do you walk or use a bicycle for at least 10 minutes continuously to get to and from places? Mention number of days.

____

How much time do you spend walking or bicycling for travel on a typical day?

____

Do you do any vigorous-intensity sports, fitness or recreational (leisure) activities that cause large increases in breathing or heart like running or football for at least 10 minute continuously?

◯ Yes
◯ No

In a typical week, on how many days do you do vigorous-intensity sports, fitness or recreational activities?

____

How much time do you spend doing vigorous-intensity sports, fitness or recreational activities on a typical day?

____

Do you do any moderate-intensity sports, fitness or recreational (leisure) activities that cause a small increase in breathing or heart rate like brisk walking, cycling, swimming, volleyball for at least 10 minutes continuously?	◯ Yes<br>◯ No
In a typical week, on how many days do you do moderate-intensity sports, fitness or recreational activities? Mention the number of days.	______________________
How much time do you spend doing moderate-intensity sports, fitness or recreational activities on a typical day?	______________________
How much time do you usually spend sitting or reclining on a typical day?	______________________

Dental health status

Please complete the survey below.

Thank you!

Have you ever had any teeth pulled out?	◯ Yes<br>◯ No<br>◯ Don't know
If yes, how many	______________________________
Why were the teeth extracted (mark all that apply)	☐ Painful tooth<br>☐ Broken tooth<br>☐ Infection<br>☐ Decayed tooth<br>☐ Loose tooth<br>☐ Wisdom tooth<br>☐ To make space for other teeth
Have you ever had your teeth cleaned by a dentist or dental hygienist? (Cleaning is also called as "scaling" or "polishing")	◯ Yes<br>◯ No<br>◯ Don't know
If yes, "How many times have you had your teeth cleaned/scaled"	◯ Once<br>◯ Occasionally<br>◯ At regular dental checkups
Do you have false teeth?	◯ Yes<br>◯ No<br>◯ Rather not say

Dental Behaviors

Please complete the survey below.

Thank you!

Have you seen a dentist before?	◯ Yes<br>◯ No<br>◯ Rather not say
If yes, when did you last see a dentist?	◯ Less than one year ago<br>◯ More than one year ago
What is your usual reason for seeing a dentist?	◯ Problem<br>◯ Check-up
Do you think you need to see a dentist?	◯ Yes<br>◯ No<br>◯ Don't know
Would you feel scared about going to the dentist?	◯ No<br>◯ A little bit<br>◯ A fair bit<br>◯ A lot
Do you have a toothbrush?	◯ Yes<br>◯ No<br>◯ Rather not say
If yes, did you brush your teeth yesterday?	◯ Yes<br>◯ No<br>◯ Don't know
If yes, did you use toothpaste?	◯ Yes<br>◯ No<br>◯ Don't know
If yes, how many times in a day do you brush your teeth?	◯ Once<br>◯ Twice<br>◯ Three times or more<br>◯ Rather not say
Do you use mouthwash?	◯ Yes<br>◯ No
How many times in a day do you use mouthwash?	◯ Once daily after brushing<br>◯ Twice daily after brushing<br>◯ Use only mouthwash once a day<br>◯ Use only mouthwash more than once a day<br>◯ Don't know/Rather not say
Do you floss or other interdental aids?	◯ Yes<br>◯ No

How many times in a day do you use floss?

- ◯ Once daily after brushing
- ◯ Twice daily after brushing
- ◯ Occasionally
- ◯ Don't know/Rather not say

Emotional wellbeing

Please complete the survey below.

Thank you!

		None of them	A little of the time	Some of the time	Most of the time	All the time
1)	During the last 30 days, about how often did you feel tired out for no good reason?	○	○	○	○	○
2)	During the last 30 days, about how often did you feel nervous?	○	○	○	○	○
3)	During the last 30 days, about how often did you feel so nervous that nothing could calm you down?	○	○	○	○	○
4)	During the last 30 days, about how often did you feel hopeless?	○	○	○	○	○
5)	During the last 30 days, about how often did you feel restless or fidgety?	○	○	○	○	○
6)	During the last 30 days, about how often did you feel so restless you could not sit still?	○	○	○	○	○
7)	During the last 30 days, about how often did you feel depressed?	○	○	○	○	○
8)	During the last 30 days, about how often did you feel that everything was an effort?	○	○	○	○	○
9)	During the last 30 days, about how often did you feel sad that nothing could cheer you up?	○	○	○	○	○
10)	During the last 30 days, about how often did you feel worthless?	○	○	○	○	○

About Survey

Please complete the survey below.

Thank you!

---

1)	How did you find out this study?	◯ Word of mouth (eg.friends, family)<br>◯ Dental clinic<br>◯ Medical or other clinic<br>◯ Advertisement<br>◯ Email<br>◯ Social media<br>◯ Newsletter
