# Supplementary material for: Development and characterization of an oral microbiome transplant among Australians for the treatment of dental caries and periodontal disease: A study protocol
Source: PLoS One. 2021 Nov 29;16(11):e0260433. doi: 10.1371/journal.pone.0260433 (PMC8629173; doi:10.1371/journal.pone.0260433)
Supplement: Attachment — Submitted filename: 0628plosone.docx [file pone.0260433.s004.docx]

This manuscript aims to develop and characterize an Oral Microbiome Transplant (OMT) for the treatment of caries and periodontal disease. Supragingival plaque samples would be collected from healthy participants to find donors suitable for microbial transplantation. Based on the current protocol, a novel in vitro flow cell model combined with microscopic examination and DNA sequencing were be used to describe the composition and functions of the donors. Hydrogels would be used to deliver microbiota for OMT. The effectiveness of OMT would be assessed using caries and periodontitis animal models. Although the current research would provide preliminary results of the feasibility of oral microbiome transplant, I still see some major areas of concern that I think need to be addressed before this manuscript is ready for primetime.

1. What is the specific definition of “super donor”? Authors mentioned that it is prudent to understand the variation and functions of a healthy oral microbiome and hence define a “super donor”, however, authors never presented data in support of any of these claims.
2. In Rodent periodontitis model, why not use silk-ligature for induction of experimental periodontitis? Also, why not include the application of a broad-spectrum antimicrobial agent before OMT?
3. “The use of hydrogel for OMT delivery provides a unique opportunity to seed the micro-organisms onto the surface of teeth for a prolonged period” Any data to support these claims?
4. Safety concerns in the application of OMT are similar to those for oral probiotics, as transplanted biofilms should possess a high degree of genetic stability. It is critical to determine whether oral biofilms should be transplanted directly from donors to patients, or pre-treated with methods that eliminate pathogenic organisms prior to transplantations. How can these problems be solved according to the current protocol?
5. In OMT, transplanted biofilms must exhibit the capacity to effectively endure the selective pressure of the environment. Why do authors choose hydrogels to deliver microbiota for OMT? Please specify in the text.
6. The discussion part should focus more on the advantages of this experimental method
