# Supplementary material for: Development and characterization of an oral microbiome transplant among Australians for the treatment of dental caries and periodontal disease: A study protocol
Source: PLoS One. 2021 Nov 29;16(11):e0260433. doi: 10.1371/journal.pone.0260433 (PMC8629173; doi:10.1371/journal.pone.0260433)
Supplement: Attachment — Submitted filename: ResponseTo Reviewer.docx [file pone.0260433.s005.docx]

We thank the reviewers for their comments. We have addressed each comment below and in the main text, and we believe that the manuscript now more clearly explains the strategies behind our approach.

The comments from the reviewer:

This manuscript aims to develop and characterize an Oral Microbiome Transplant (OMT) for the treatment of caries and periodontal disease. Supragingival plaque samples would be collected from healthy participants to find donors suitable for microbial transplantation. Based on the current protocol, a novel in vitro flow cell model combined with microscopic examination and DNA sequencing were be used to describe the composition and functions of the donors. Hydrogels would be used to deliver microbiota for OMT. The effectiveness of OMT would be assessed using caries and periodontitis animal models. Although the current research would provide preliminary results of the feasibility of oral microbiome transplant, I still see some major areas of concern that I think need to be addressed before this manuscript is ready for primetime.

**1.** What is the specific definition of “super donor”? Authors mentioned that it is prudent to understand the variation and functions of a healthy oral microbiome and hence define a “super donor”, however, authors never presented data in support of any of these claims.

 Author’s response: This concept is borrowed from fecal microbiota transplant literature (reviewed in Wilson, et al. Front. Cell. Infect. Microbiol., 2019). An oral microbiota ‘super donor’ is now defined in the text in the context of this approach and discussed in great detail in the introduction (page 8, Line 169-190) and discussion (page 22, line 486-488, 496-508).

**2.** In Rodent periodontitis model, why not use silk-ligature for induction of experimental periodontitis? Also, why not include the application of a broad-spectrum antimicrobial agent before OMT?

 Author’s response: This is a wonderful suggestion. We now provide a rational as to why this approach was not selected, but it would be useful in the exploration of OMT efficacy for a multitude of reasons. We have now included it in the discussion (page 24, line 550-562).

**3.** “The use of hydrogel for OMT delivery provides a unique opportunity to seed the micro-organisms onto the surface of teeth for a prolonged period” Any data to support these claims?

 Author’s response: As OMT therapy has not yet been delivered yet in humans (which we now make clear in the introduction of the paper), there are not any references to support this claim. Therefore, we have clarified our statement and provided further rational to support the selection of hydrogel developed in OMTs (page 23-24, line 529-546).

**4.** Safety concerns in the application of OMT are similar to those for oral probiotics, as transplanted biofilms should possess a high degree of genetic stability. It is critical to determine whether oral biofilms should be transplanted directly from donors to patients, or pre-treated with methods that eliminate pathogenic organisms prior to transplantations. How can these problems be solved according to the current protocol?

 Author’s response: We agree that this is a critical issue. We have added a brief discussion of this into manuscript in the context of our *in vivo* model (Page 19, line 432-434, and page 20, line 458-462), although directly testing this will likely require early clinical trials in humans. In murine models for caries and periodontitis, prior to application of OMT, full mouth debridement and disinfection would be carried out. For safety assessments in murine models, discomfort would be measured by vital parameters and any adverse reactions (page 20-21, line 477-479).

**5.** In OMT, transplanted biofilms must exhibit the capacity to effectively endure the selective pressure of the environment. Why do authors choose hydrogels to deliver microbiota for OMT? Please specify in the text.

 Author’s response: We provide an improved rational for why hydrogels were selected in the discussion ( Page 23-24, line 523-546) and also include a brief discussion of other methods that could be employed, if hydrogels fail (Page 16). We will test the efficacy of delivering OMT in hydrogels in murine models. In addition, other modes of delivery would also be tested such as buffers, varnishes and mouthwash (page 16-17, line 383-386).

6. The discussion part should focus more on the advantages of this experimental method.

Author’s response: We have completely revamped the discussion to include significant discussion of the advantages of this approach (Page 212, line 496-508, page 23 line 520-522, line 523-527, line 540-546, 563-564).
